# Comprehensive Evaluation of Leaf Structure, Photosynthetic Characteristics, and Drought Resistance in Six Jackfruit (*Artocarpus heterophyllus*) Cultivars

**DOI:** 10.3390/life15091346

**Published:** 2025-08-26

**Authors:** Weihao Wu, Chongcheng Yang, Shiting Lin, Wei Li, Suhui Ou, Jinson Guo, Xiaojia Huang, Xuemin Liu, Feng Feng

**Affiliations:** 1 College of Coastal Agricultural Sciences, Guangdong Ocean University, Zhanjiang 524088, China; 11321122aa@stu.gdou.edu.cn (W.W.); 15014960216@stu.gdou.edu.cn (C.Y.); linjianqiang@stu.gdou.edu.cn (S.L.); 17820335211@163.com (W.L.); ousuhui@stu.gdou.edu.cn (S.O.); 17506063695@163.com (J.G.); 11321109dd@stu.gdou.edu.cn (X.H.); 2 Honghe Tropical Agriculture Institute of Yunnan, Mengzi 661100, China

**Keywords:** jackfruit (*Artocarpus heterophyllus* Lam), leaf structure, photosynthetic characteristics, drought resistance evaluation

## Abstract

Drought stress is one of the key abiotic stress factors limiting the growth and development, yield formation, and improvement in the quality of jackfruit (*Artocarpus heterophyllus*). However, systematic evaluations of drought tolerance in jackfruit germplasm resources remain limited. In this study, six jackfruit cultivars were used as materials. By systematically comparing 26 indicators, including leaf structural characteristics, chlorophyll concentration, and photosynthetic parameters, the primary evaluation indicators for jackfruit drought tolerance were identified, and clear microscopic structural images of leaves from different jackfruit cultivars were obtained. In this study, significant differences were observed among different jackfruit germplasm resources in terms of leaf structure, chlorophyll concentration, and photosynthetic characteristics. Comprehensive analysis identified *A.* ‘Changyou’ as the jackfruit cultivar with the highest drought tolerance score and *A*. ‘Siji’ as the variety with the lowest drought tolerance score. By establishing a systematic evaluation system for jackfruit drought tolerance, it was found that jackfruit cultivars with high drought tolerance had significantly thicker palisade parenchyma than other cultivars, a rougher leaf epidermis, and more densely distributed stomata on the leaves, while their chlorophyll concentration was significantly lower than that of cultivars with lower drought tolerance scores. Jackfruit cultivars with the lowest drought resistance scores had significantly lower net photosynthetic rates, transpiration rates, stomatal conductance, and light saturation points than other cultivars. This study’s results established a drought resistance evaluation system for jackfruit germplasm resources, providing theoretical support for the selection and breeding of high-drought-resistant superior jackfruit cultivars.

## 1. Introduction

Jackfruit (*Artocarpus heterophyllus* Lam) is a typical tropical fruit tree of the genus *Artocarpus* of the family Moraceae, native to the rainforests of the Western Ghats of India [1]. Jackfruit is widely cultivated in tropical and subtropical regions around the world, particularly in Southeast Asia, South Asia, and China’s Hainan, Guangdong, Guangxi, and Yunnan Provinces, due to its unique flavor, rich nutritional value, and enormous economic potential [2]. Jackfruit is the world’s largest fruit, its mature pulp and seeds are edible and they have a medicinal value, and the wood also has a high economic value, so it plays an important role for farmers as producing the fruit can increase their income and economic development [3]. With the exchange and dissemination of jackfruit germplasm, many jackfruit germplasm resources have been introduced and preserved in the Guangdong Jackfruit Germplasm Resource Nursery. However, there has been a lack of systematic research and evaluation of the drought resistance of these jackfruit resources.

Drought is one of the primary abiotic stresses limiting plant growth and crop production in many regions [4]. Plant adaptability and tolerance to drought stress constitute a complex, multifaceted trait closely associated with their morphological structure and physiological and biochemical processes [5]. Plants typically develop various drought-resistant morphologies to cope with drought stress [6]. Among these, leaves serve as the primary sites for water transport, material exchange, and photosynthesis in plants. Additionally, their morphological and anatomical structures are crucial for plant taxonomic classification, evolutionary studies, and research into environmental adaptation capabilities [7,8].

When evaluating the drought tolerance of fruit tree resources, researchers typically compare the leaf anatomical structure and photosynthetic characteristics among varieties, using principal component analysis and membership function evaluation methods [9,10]. Li et al. (2025) compared the microscopic leaf structures of 96 apricot germplasm resources and identified leaf thickness, stomatal length, palisade parenchyma thickness, stomatal width, stomatal area, upper epidermal cell thickness, and upper epidermal cell width as effective drought resistance evaluation indicators [7]. Lei et al. (2025) compared the leaf anatomical structure characteristics of 14 mango germplasm resources and found that mango resources with stronger drought resistance had thicker leaves, main veins, upper and lower epidermal cells, and upper and lower cuticle layers, as well as higher palisade-mesophyll ratios and leaf parenchyma structural density [11]. Ma et al. (2023) studied the leaf anatomical structure of *Macadamia* germplasm resources and evaluated their drought resistance, concluding that *Macadamia* cultivars with thick leaves, dense palisade parenchyma, loose spongy parenchyma, and closely arranged main veins and vascular bundles exhibit better drought resistance [12].

As a tropical fruit tree, jackfruit has a certain heat tolerance. However, prolonged or severe drought stress can significantly inhibit its photosynthetic efficiency, disrupt cellular structure, and hinder nutrient transport, ultimately leading to weakened tree vigor, flower and fruit drop, reduced fruit quality, and even plant death, severely constraining the sustainable development of the industry [13,14]. Current research on jackfruit primarily focuses on cultivation techniques, pest and disease control, post-harvest physiology of fruits, and preliminary evaluations of certain cultivars [15,16]. In-depth studies on the microscopic structural differences among different jackfruit cultivars and their systematic correlations with photosynthetic performance and drought tolerance remain unexplored, and a comprehensive drought tolerance evaluation system is currently lacking.

In this study, we used six jackfruit cultivars as experimental materials to compare the morphological characteristics, key anatomical structures, chlorophyll concentration, and photosynthetic characteristics of the leaves of each cultivar. Finally, we evaluated drought resistance based on a comprehensive set of relevant indicators, with the aim of providing a reference for the innovative use of drought-resistant jackfruit materials.

## 2. Materials and Methods

### 2.1. Experimental Materials

The test materials were obtained from *A*. ‘Haida 1’, *A*. ‘Haida 2’, *A.* ‘Haida 3’, *A*. ‘Haida 4’, *A*. ‘Siji’, and *A*. ‘Changyou’, which are 6 cultivars of jackfruit grown in the Jackfruit Germplasm Resource Nursery of Guangdong Province (110°30′ E, 21°16′ N). The soil of the nursery was deep, brick red soil: soil pH: 5.0–5.6, soil capacity: 1.36 g/cm^3^, soil organic carbon: 14.36–16.04 g/kg, total nitrogen: 1.23–1.31 g/kg, ammonium nitrogen: 25.69–26.71 mg/kg, nitrate nitrogen: 1.74–2.04 mg/kg, and available phosphorus: 67.46–75.14 mg/kg. The annual sunshine hours are 1915 h, the annual precipitation is 1439.9 mm, and the average annual temperature is 23.2 °C. The test materials were all grafted seedlings, and the rootstocks were *A*. ‘Malaysia 1’ seedlings (Table 1). The plants were planted in May 2012, in red soil, with 10 plants of each variety, sequentially arranged, with a spacing of 6 m × 6 m between rows.

### 2.2. Experimental Methods

#### 2.2.1. Methods for Sampling Plant Leaves

The methods for selecting leaf samples used in the experiments for leaf characteristic analysis, anatomical structure observation, chlorophyll concentration determination, and photosynthetic index measurement were consistent. Specifically, three healthy plants were selected from each variety, and branches without pests or diseases were randomly selected from the sunny side of the plant. The fourth or fifth leaf from the top, which was of a similar size, was selected as the fresh leaf sample.

#### 2.2.2. Observation of Leaf Morphological Characteristics

Samples were taken on 7 August 2024, from 9:00 to 11:00 a.m. Leaves were selected according to the plant leaf sampling method. Leaf length, leaf width, leaf shape index, and leaf area were determined using Image-Pro Plus 6.0 image processing and analyzing software, and each leaf was replicated five times. Leaf shape index = blade length/blade width [18]. A leaf microstructure observation was carried out by field emission scanning electron microscope–energy dispersive spectrometer (TESCAN MIRA 3 LMU, EDAX, Czech Republic) scanning as follows: Take small square areas (1 cm × 1 cm) from the midrib of leaf samples from different jackfruit cultivars. Fix the leaf samples in FAA fixative for 24 h, and then remove them. Rinse the samples once every 15 min with 0.1 mol/L of phosphate-buffered solution for a total of three rinses. Perform dehydration using a gradient ethanol solution at concentrations of 30%, 50%, 70%, 80%, 90%, and 100%, each for 15 min, followed by the replacement of 100% ethanol with tert-butanol. At room temperature, first replace with an ethanol–tert-butanol solution for 20 min, then replace with 100% tert-butanol for 30 min, followed by one additional replacement with 100% tert-butanol. Dry the samples using natural drying; attach the samples to conductive carbon film double-sided tape and place them on the ion sputtering instrument sample stage for 120 s of gold sputtering for conductivity treatment; observe and record images of the samples under a scanning electron microscope at an acceleration voltage of 10 kV; measure the pore density and size; and, finally, repeat the process three times for each material [19]. The individual stomatal area (S) was calculated according to the following equation [20]:

Area of individual stomata (S) = a × b × π × 1/4(1)

In the formula, the base unit of the stomatal density is one/mm^2^, a is the longitudinal axis of the stomatal apparatus, b is the transverse axis of the stomatal apparatus, and the base unit of the individual stomatal area is μm^2^.

#### 2.2.3. Measurement of Leaf Anatomy

Leaf anatomy was analyzed by paraffin sectioning [21]. Leaves were selected according to the leaf sampling method described in 2.2.1. Small square areas (1 cm × 1 cm) were cut from the midribs of leaves from six jackfruit varieties to prepare 8 mm thick paraffin sections. These sections were then observed and photographed using an ECLIPSE Ci-L biological microscope (Nikon, Shanghai, China), with three random fields of view observed for each sample. Photographs were taken using an Eclipse Ci-L microscope and analyzed using Image-Pro Plus 6.0 software to determine leaf thickness (LT), thickness of upper epidermal cells (TU), thickness of lower epidermal cells (TL), thickness of the upper epidermal thick-walled parenchyma (UC), lower epidermal thick-walled parenchyma thickness (LC), palisade parenchyma thickness (TP), sponge parenchyma thickness (TS), thick wall thickness (TW), and vessel diameter (VD). We also calculated the parenchyma ratio of palisade to spongy (palisade parenchyma thickness/sponge parenchyma thickness), leaf parenchyma structure tightness CTR (palisade parenchyma thickness/leaf thickness × 100%), and spongy mesophyll ratio SR (sponge parenchyma thickness/leaf thickness × 100%).

#### 2.2.4. Determination of Leaf Chlorophyll Concentration

Chlorophyll concentration was determined by the ethanol–acetone mixture extraction method. The fresh leaves of the collected samples were rinsed clean and dried with deionized water, avoiding the leaf veins, cut into 2 mm thin strips, mixed well, divided into a 0.1 g per portion, repeated 3 times, put into 10 mL corked test tubes, had 10 mL of 95% ethanol–acetone mixed extract with a volume ratio of 1:1 added, and immersed in darkness for 48 h to be whitened by all the leaf parenchyma. The mixed extract was used as a control, 100% transmittance was adjusted, and the absorbance values at wavelengths of 663, 645, and 470 nm were determined by a UV–visible spectrophotometer (UV-5500PC, Shanghai Metash Instruments Co., Ltd., Shanghai, China). The chlorophyll concentration was calculated using the following formula:

Chlorophyll a content (mg/g^−1^): Ca = (12.7A663 − 2.69A645) × V/(1000 × W)

Chlorophyll b content (mg/g^−1^): Cb = (22.9A645 − 2.69A663) × V/(1000 × W)

Total chlorophyll concentration C = Ca + Cb

where V was the volume of the extracted liquid (mL); W was the fresh weight of the sample (g).

#### 2.2.5. Determination of Leaf Photosynthetic Indexes

On 7 August 2024, from 6:00 AM to 7:00 PM, measurements were conducted on intact leaves with consistent conditions regarding location, height, and orientation using a plant photosynthesis measurement instrument (TP-3051D, Hangzhou, Zhejiang, China). We measured the net photosynthesis rate (Pn), transpiration rate (Tr), stomatal conductance (Gs), and intercellular CO_2_ concentration (Ci) of mature leaves from six cultivars of jackfruit. Measurements were taken every hour, totaling 12 measurements, with three replicates per variety. Data on the effect of light intensity on the net photosynthetic rate (Pn) of leaves were analyzed and curve-fitted using the ‘logarithmic’, ‘quadratic’, and ‘cubic’ terms of SPSS 26.0 curve estimation. Curve fitting was performed to determine the best-fitting model based on *p*-values and *R*^2^ values and quantify the effect of light intensity. The fitted equations were used to calculate the light saturation point and light compensation point of different jackfruit leaves.

### 2.3. Statistics and Analysis of Data

The test results were statistically analyzed and graphed using Excel 2010, SPSS 25.0, and GraphPad Prism 10 software, and the significance of the differences in each index between treatments was tested using a one-way ANOVA (one-way ANOVA) and the least significant difference method (SSR). Pearson’s method was used to perform a two-tailed test of the correlation among the morphological indexes, leaf anatomical structure indexes, and chlorophyll and photosynthetic indexes. The drought tolerance values of the six species of jackfruit were calculated and ranked by principal component analysis, the subordinate function method, and principal component weighting method.

Firstly, the data of each index were standardized and subjected to principal component analysis, and the principal components were extracted according to the principle that the characteristic root was greater than 1 and the cumulative contribution rate was greater than 85%. The coefficients of each principal component were calculated according to Formula (1), and the score value of the principal component of each tree species was calculated by constructing the linear expression between each principal component and each index (C). The affiliation function value (U) of the principal component score value of each tree species was calculated according to Formula (2). According to the size of the contribution rate of the principal components, Formula (3) was used to calculate the corresponding weights (Wi), and the comprehensive evaluation value of drought resistance of each species (D) was calculated according to Formula (4).

(2) $$t_{i}=a_{i}∕\sqrt{\lambda i}\left(i=1,2,\ldots ,n\right)$$

where t_i_ is the coefficient of the ith principal component factor, λi is the loading vector of the ith principal component factor, and λi is the eigenroot of the ith principal component factor.

(3) $$U_{i}=\left(X_{i}-X_{\min}\right)∕\left(X_{\max}-X_{\min}\right)$$

where U_i_ is the value of the affiliation function of the score value of the ith principal component, X_i_ is the score value of the ith principal component, X_min_ is the minimum value of the score value of the ith principal component, and X_max_ is the maximum value of the score value of the ith principal component.

(4) $$W_{i}=P_{i}∕\sum_{i=1}^{n}P_{i}\left(i=1,2,\ldots ,n\right)$$

(5) $$D=\sum_{i=1}^{n}\left(U_{i}\times W_{i}\right)$$

where W_i_ is the weight of the ith composite assessment value among all composite assessment values, P_i_ is the contribution rate of the ith composite assessment value, and D is the composite assessment value.

## 3. Results and Analysis

### 3.1. Analysis of the Leaf Structure of Different Strains of Jackfruit

#### 3.1.1. Morphological Characteristics of the Leaf Blades of Different Strains of Jackfruit

**Figure 1:**
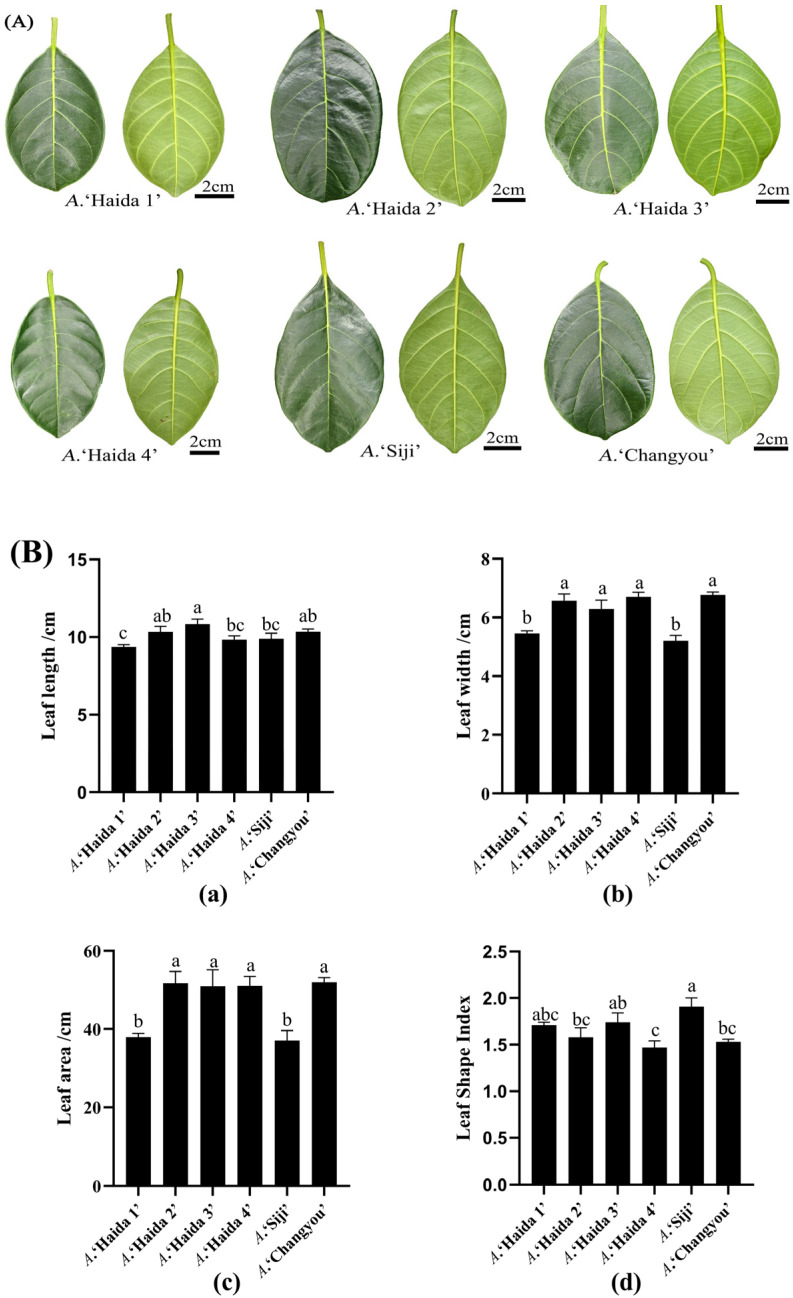
Morphological characteristics of leaf blades of different Bolognese cultivars: (**A**) leaf phenotype; (**B**) leaf index; (**a**) leaf length; (**b**) leaf width; (**c**) leaf area; (**d**) leaf shape index. Different lowercase letters indicate significant differences (*p* < 0.05).

Leaf width, leaf length, leaf area, and leaf shape index were measured for the leaves of six cultivars of jackfruit, and the results showed that there were significant differences in leaf size and morphological characteristics among the six cultivars of jackfruit (Figure 1). Among them, *A*. ‘Haida 3’ had the longest leaf blade and *A*. ‘Haida 1’ had the shortest leaf blade; *A*. ‘Haida 1’ and *A*. ‘Siji’ had significantly smaller leaf widths and leaf areas than the other jackfruit cultivars; and *A*. ‘Siji’ had a significantly higher leaf shape index than that of *A*. ‘Haida 2’, *A*. ‘Haida 4’, and *A*. ‘Changyou’.

#### 3.1.2. Stomatal Size of Leaves of Different Strains of Jackfruit

**Figure 2:**
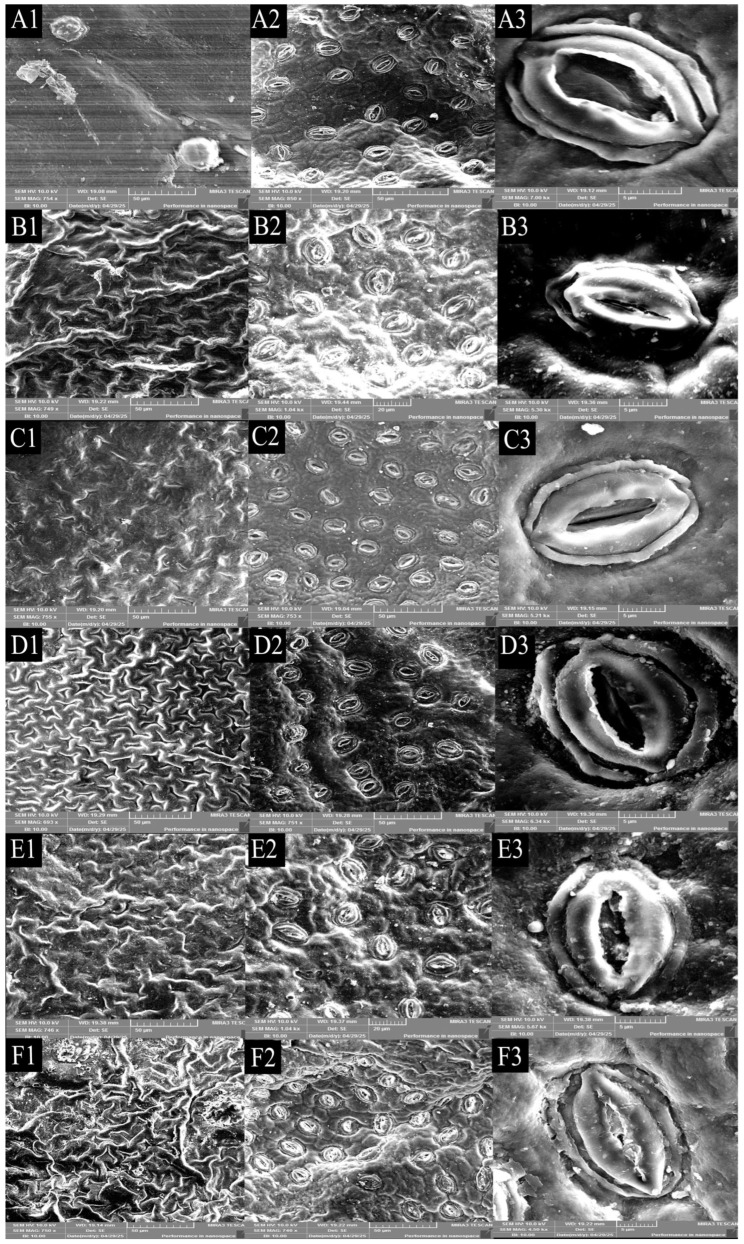
Electron microscope scanning and stomata of leaves of different jackfruit cultivars. (**A**) *A*. ‘Haida 1’; (**B**) *A*. ‘Haida 2’; (**C**) *A*. ‘Haida 3’; (**D**) *A*. ‘Haida 4’; (**E**) *A*. ‘Siji’; (**F**) *A*. ‘Changyou’. 1: Upper epidermal electron microscopy; 2: lower epidermal electron microscopy; 3: stomata.

**Figure 3:**
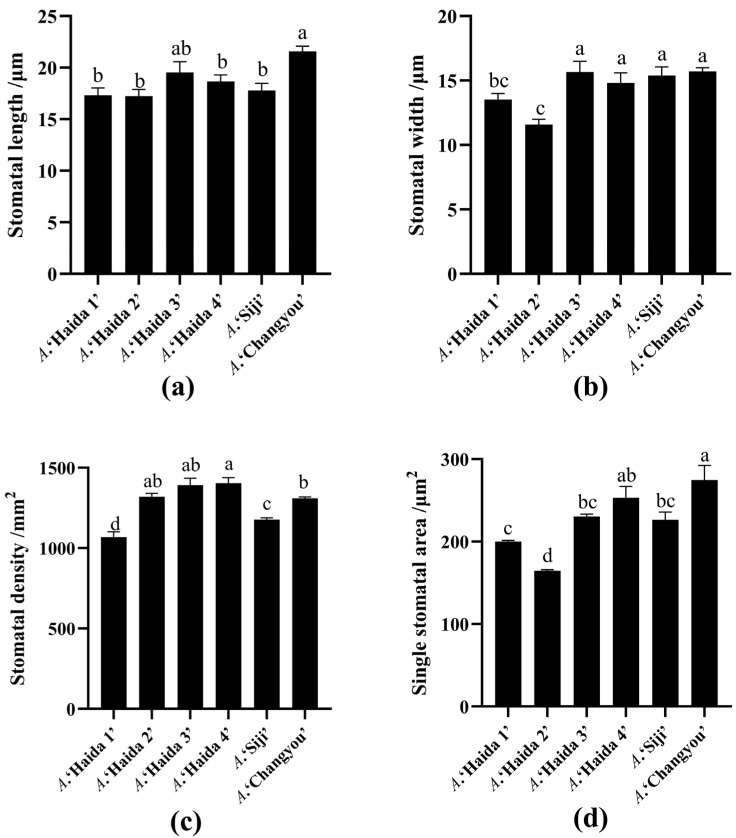
Comparison of the size of stomata in different cultivars of jackfruit. (**a**) Stomatal length; (**b**) stomatal width; (**c**) stomatal density; (**d**) area of a single stomatal pore. Different lowercase letters indicate significant differences (*p* < 0.05).

The electron microscope scanning pictures of the leaves of different jackfruit strains are shown in Figure 2, and it can be clearly found that the epidermal texture on the leaves of *A*. ‘Haida 1’ was the smoothest, and the epidermal texture folds on the leaves of *A*. ‘Haida 4’ and *A*. ‘Changyou’ are relatively clear and regular; the difference in the epidermis on the leaves can be used as a structural feature to differentiate between the cultivars of jackfruit. There were significant differences in the stomatal morphology characteristics among the different jackfruit strains (Figure 3). *A*. ‘Changyou’ was significantly longer than *A*. ‘Haida 1’, *A*. ‘Haida 2’, *A*. ‘Haida 4’, and *A*. ‘Siji’; the stomata of *A*. ‘Haida 2’ were the narrowest; and the stomata of *A*. ‘Haida 1’ and *A*. ‘Haida 2’ were significantly wider than those of the other cultivars. There was no significant difference in the stomatal density of *A*. ‘Haida 2’, *A*. ‘Haida 3’, and *A*. ‘Haida 4’. The stomatal density of *A*. ‘Haida 4’ was significantly greater than that of *A*. ‘Changyou’, *A*. ‘Siji’, and *A*. ‘Haida 1’, while the stomatal density of *A*. ‘Haida 1’ was the lowest and significantly less than that of the other cultivars. The stomatal area of *A*. ‘Changyou’ was the largest and significantly greater than that of *A*. ‘Siji’, *A*. ‘Haida 3’, *A*. ‘Haida 1’, and *A*. ‘Haida 2’, of which *A*. ‘Haida 2’ was the smallest and significantly smaller than the other cultivars.

#### 3.1.3. Anatomical Structure of the Leaf Blades of Different Strains of Jackfruit

**Figure 4:**
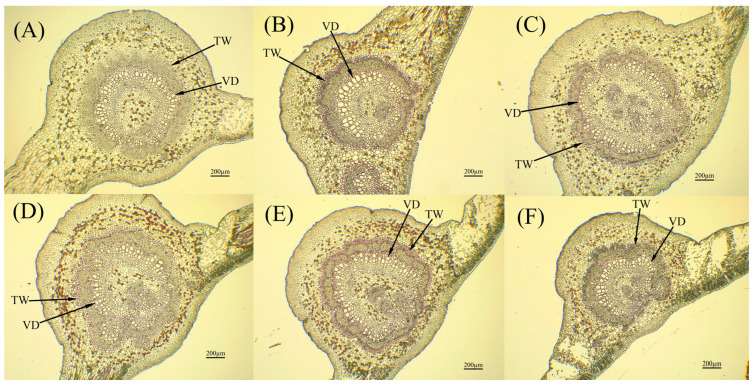
Anatomical structure of leaf vein sections of different jackfruit cultivars. (**A**) *A*. ‘Haida 1’; (**B**) *A*. ‘Haida 2’; (**C**) *A*. ‘Haida 3’; (**D**) *A*. ‘Haida 4’; (**E**) *A*. ‘Siji’; (**F**) *A*. ‘Changyou’. TW: Thick wall thickness; VD: vessel diameter.

**Figure 5:**
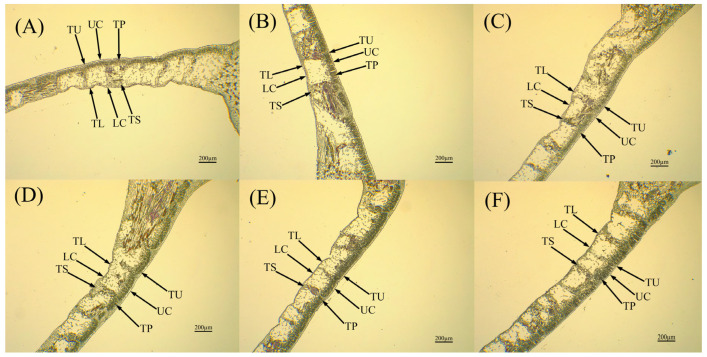
Anatomical structure of leaf flesh slices of different jackfruit cultivars: (**A**) *A*. ‘Haida 1’; (**B**) *A*. ‘Haida 2’; (**C**) *A*. ‘Haida 3’; (**D**) *A*. ‘Haida 4’; (**E**) *A*. ‘Siji’; (**F**) *A*. ‘Changyou’. TU: thickness of upper epidermal cells, TL: thickness of lower epidermal cells, UC: thickness of the upper epidermal thick-walled parenchyma, LC: lower epidermal thick-walled parenchyma thickness, TP: palisade parenchyma thickness, TS: sponge parenchyma thickness.

The leaf structures of different cultivars of jackfruit were obtained by paraffin sections (Figure 4 and Figure 5), and the results showed that there were significant differences in the leaf structures of different strains of jackfruit (Supplementary Table S1). The leaf thicknesses of *A*. ‘Haida 4’, *A*. ‘Haida 1’, *A*. ‘Changyou’, and *A*. ‘Haida 3’ were significantly greater than those of *A*. ‘Siji’ and *A*. ‘Haida 2’, while *A*. ‘Siji’ had the thinnest leaves, which were highly significantly lower than those of the other cultivars. *A*. ‘Haida 2’ had the largest vessel diameter, but it was not significantly different from *A*. ‘Haida 1’, *A*. ‘Haida 3’, or *A*. ‘Siji’ and was highly significantly larger than that of *A*. ‘Haida 4’ and *A*. ‘Changyou’. There were significant differences in the parenchyma ratio of palisade to spongy of different strains of jackfruit: *A*. ‘Changyou’ > *A*. ‘Siji’ > *A*. ‘Haida 2’ > *A*. ‘Haida 3’ > *A*. ‘Haida 4’ > *A*. ‘Haida 1’. The leaf parenchyma structure of *A*. ‘Changyou’ and *A*. ‘Siji’ was significantly larger than that of the other cultivars, and the leaf parenchyma structure of *A*. ‘Haida 1’ was the least compact. The leaf parenchyma structure of *A*. ‘Haida 4’ was the most lax and significantly larger than that of the other cultivars, while there were no significant differences between *A*. ‘Siji’ and *A*. ‘Haida 2’, and no highly significant differences between *A*. ‘Haida 1’, *A*. ‘Changyou’, and *A*. ‘Haida 3’.

### 3.2. Comparison of Chlorophyll Concentration of Different Strains of Jackfruit

**Figure 6:**
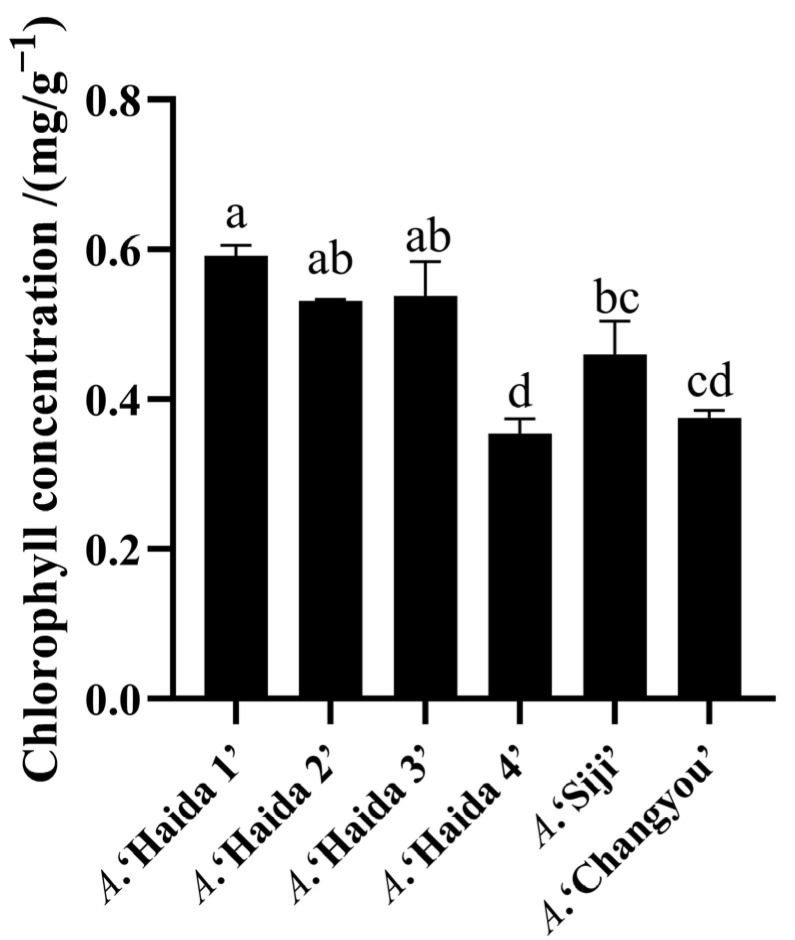
Chlorophyll concentration of different jackfruit cultivars. Different lowercase letters indicate significant differences (*p* < 0.05).

The chlorophyll concentration of different jackfruit cultivars is shown in Figure 6, and there were significant differences in the chlorophyll concentration among the different jackfruit cultivars. *A*. ‘Haida 1’ had the highest chlorophyll concentration and *A*. ‘Haida 4’ had the lowest chlorophyll concentration; the chlorophyll concentrations were, in descending order, *A*. ‘Haida 1’, *A*. ‘Haida 3’, *A*. ‘Haida 2’, *A*. ‘Siji’, *A*. ‘Changyou’, and *A*. ‘Haida 4’.

### 3.3. Comparison of Photosynthetic Characteristics of Different Strains of Jackfruit

#### 3.3.1. Analysis of Daily Variation in Photosynthesis in Different Strains of Jackfruit

The daily variation in the net photosynthetic rate of different jackfruit cultivars is shown in Supplementary Figure S1, and it can be seen that different jackfruit cultivars showed different significant differences in the net photosynthetic rate. However, except for *A*. ‘Siji’, the rate mainly increased significantly to reach its peak in the morning, decreased to noon, and then increased significantly in the afternoon and then decreased significantly, showing an M-type net photosynthetic rate curve. The net photosynthetic rate of *A*. ‘Siji’ was the lowest among the six jackfruit cultivars, although it had three peaks. The daily variation in the transpiration rate of different jackfruit cultivars is shown in Supplementary Figure S2. The results showed that the peak of the transpiration rate of different jackfruit cultivars mainly occurred at 9:00–11:00 a.m., with multiple peaks in *A*. ‘Haida 1’, *A*. ‘Haida 2’, and *A*. ‘Siji’; the overall transpiration rates of *A*. ‘Haida 1’ and *A*. ‘Siji’ were lower than those of the other cultivars. The daily changes in the stomatal conductance of different jackfruit cultivars are shown in Supplementary Figure S3. The results showed that *A*. ‘Haida 2’, *A*. ‘Haida 3’, and *A*. ‘Haida 4’ had similar trends, with the peaks occurring at 9:00–11:00 a.m.; *A*. ‘Haida 1’ and *A*. ‘Changyou’ had similar trends in the daily changes in stomatal conductance and showed multi-peak fluctuation trends; the daily changes in stomatal conductance in four-season Polaris showed an overall upward and then downward trend, with peaks at 11:00 a.m.; and the overall rate of change in the stomatal conductance of *A*. ‘Siji’ was lower than that of other cultivars. The daily variation in the intercellular CO_2_ concentration content of different jackfruit cultivars is shown in Supplementary Figure S4. The intercellular CO_2_ of different jackfruit cultivars varied significantly, with a general trend of a peak in the morning followed by a decline, and then a rise in the afternoon followed by a decline.

#### 3.3.2. Analysis of Light Intensity Response of Leaves of Different Strains of Jackfruit

**Figure 7:**
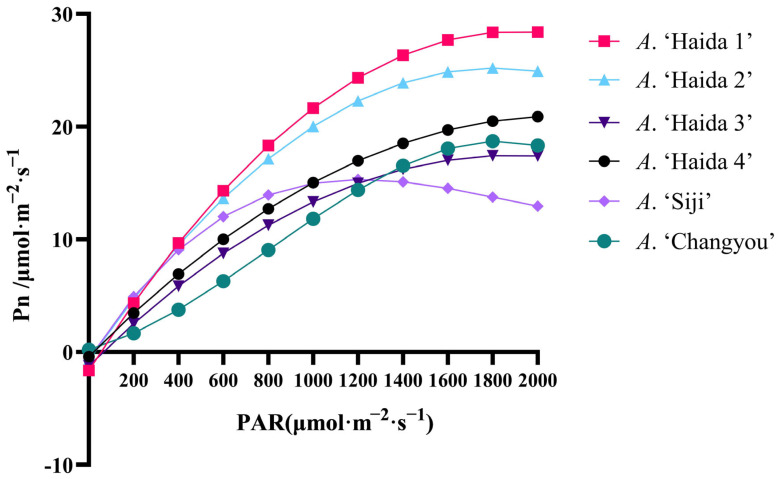
Analysis of photosynthesis–light intensity response of jackfruit leaves from different cultivars.

The corresponding change curves of the light intensity of different jackfruit leaves were obtained by fitting the equations (Figure 7). Under a normal variation in the light intensity, the net photosynthetic rate of each variety gradually increased with increasing light intensity. The light saturation points of different jackfruit cultivars were *A*. ‘Haida 1’ > *A*. ‘Haida 2’ > *A*. ‘Haida 4’ > *A*. ‘Changyou’ > *A*. ‘Haida 3’ > *A*. ‘Siji’ under the variation in light intensity of 2000 μmol-m^−2^-s^−1^. The light compensation points of different Pollosia cultivars were different: *A*. ‘Haida 3’ > *A*. ‘Haida 1’ > *A*. ‘Haida 2’ > *A*. ‘Haida 4’ > *A*. ‘Siji’ > *A*. ‘Changyou’. *A*. ‘Siji’ reached the light saturation point under the change in light intensity of 1200 μmol-m^−2^-s^−1^, which occurred earlier than that of all the other cultivars to reach the saturation point.

### 3.4. Evaluation of Drought Tolerance of Different Cultivars of Jackfruit

#### 3.4.1. Correlation Analysis of Jackfruit Leaf Indicators

**Figure 8:**
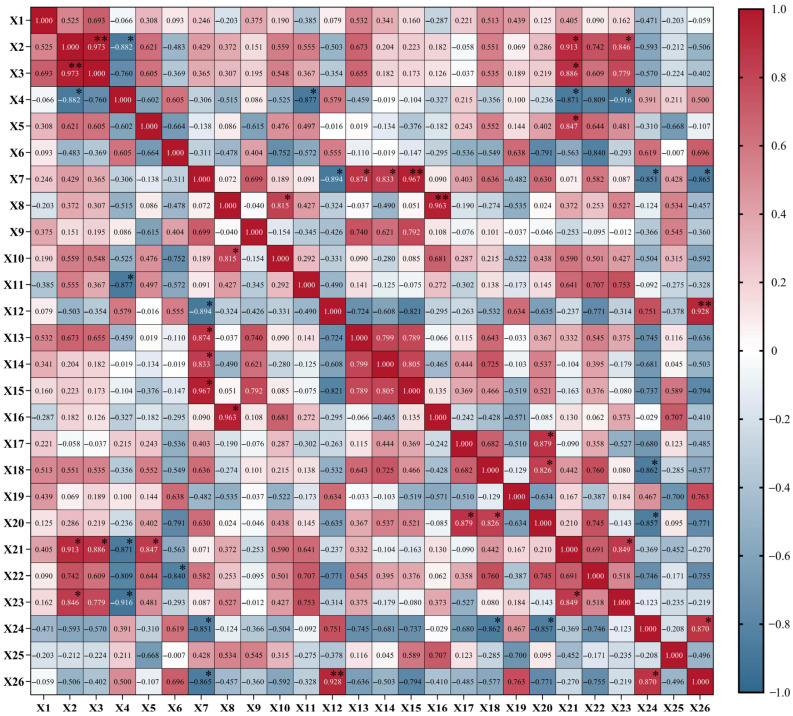
Correlation analysis of leaf indicators among different cultivars of jackfruit. X1: Leaf length; X2: leaf width; X3: leaf area; X4: leaf shape index; X5: leaf thickness; X6: vessel diameter; X7: palisade parenchyma thickness; X8: spongy parenchyma thickness; X9: thick wall thickness; X10: thickness of upper epidermal cells; X11: thickness of the upper epidermal thick-walled parenchyma; X12: thickness of lower epidermal cells; X13: lower epidermal thick-walled parenchyma thickness; X14: parenchyma ratio of palisade to spongy; X15: cell tightness ratio; X16: spongy mesophyll ratio; X17: stomatal width; X18: stomatal length; X19: stomatal density; X20: individual stomatal area; X21: net photosynthetic rate; X22: transpiration rate; X23: stomatal conductance; X24: intercellular CO_2_ concentration; X25: instantaneous water utilization; X26: chlorophyll concentration. *: significant correlation (*p* < 0.05); **: highly significant correlation (*p* < 0.01).

Correlation analysis was performed on 26 indexes such as leaf morphological characteristics, anatomical structure, and the chlorophyll and photosynthetic characteristics of six jackfruit cultivars, and the results are shown in Figure 8. Leaf width was highly significantly and positively correlated with leaf area, significantly and positively correlated with the net photosynthetic rate and stomatal conductance, and significantly and negatively correlated with the leaf shape index. Leaf area was significantly and positively correlated with the net photosynthetic rate. The leaf shape index was significantly and negatively correlated with the thickness of the upper epidermal thick-walled parenchyma, net photosynthetic rate, and stomatal conductance. Leaf thickness was significantly and positively correlated with the net photosynthetic rate. Vessel diameter was significantly negatively correlated with the transpiration rate. Palisade parenchyma thickness was significantly negatively correlated with the thickness of lower epidermal cells, intercellular CO_2_ concentration, and chlorophyll concentration and significantly positively correlated with lower epidermal thick-walled parenchyma thickness, the parenchyma ratio of palisade to spongy, and the cell tightness ratio. The sponge parenchyma thickness was significantly positively correlated with the thickness of upper epidermal cells and the spongy mesophyll ratio. The thickness of lower epidermal cells showed a highly significant positive correlation with the chlorophyll concentration. Stomatal width showed a significant positive correlation with individual stomatal area; stomatal length showed a significant positive correlation with individual stomatal area and a significant negative correlation with the interstitial CO_2_ concentration; and individual stomatal area showed a significant negative correlation with the interstitial CO_2_ concentration. The net photosynthetic rate showed a significant positive correlation with stomatal conductance; the interstitial CO_2_ concentration showed a significant positive correlation with the chlorophyll concentration.

Principal component analysis was performed on 26 indexes of the leaves of six jackfruit cultivars, and the results are shown in Table 2. According to the principle that the characteristic root is greater than 1 and the cumulative contribution rate is greater than 85%, four principal components were extracted, which are represented by C1, C2, C3, and C4. The cumulative contribution rate reached 92.96%, indicating that these four principal components could represent the main information of the original indexes, and the original 26 individual indexes were converted into 4 comprehensive indexes that were independent of each other. The higher principal component coefficients of leaf width, thickness of lower epidermal cells, transpiration rate, and chlorophyll concentration for the first principal component indicated that the first principal component was mainly determined by these four factors. The higher principal component coefficients of cell tightness ratio, net photosynthetic rate, and stomatal conductance for the second principal component indicated that the second principal component was mainly determined by these three factors. The higher principal component coefficients of spongy parenchyma thickness, spongy mesophyll ratio, stomatal density, and instantaneous water utilization of the third principal component indicated that the third principal component was mainly determined by these four factors. The higher principal component coefficients of thick wall thickness and stomatal width of the fourth principal component indicated that the fourth principal component was mainly determined by these two factors.

#### 3.4.2. Comprehensive Evaluation of Drought Tolerance

Linear expressions between each principal component and each indicator were constructed based on the coefficients of each principal component in Table 2:

C1 = 0.309X1 + 0.809X2 + 0.725X3 − 0.743X4 + 0.462X5 − 0.748X6 + 0.774X7 + 0.373X8 + 0.23X9 + 0.625X10 + 0.488X11 − 0.846X12 + 0.735X13 + 0.489X14 + 0.608X15 + 0.228X16 + 0.394X17 + 0.757X18 − 0.452X19 + 0.74X20 + 0.636X21 + 0.916X22 + 0.5X23 − 0.902X24 + 0.115X25 − 0.896X26

C2 = −0.037X1 + 0.432X2 + 0.407X3 − 0.582X4 + 0.677X5 − 0.258X6 − 0.586X7 + 0.375X8 − 0.652X9 + 0.341X10 + 0.607X11 + 0.3X12 − 0.327X13 − 0.688X14 − 0.739X15 + 0.19X16 − 0.478X17 − 0.19X18 + 0.357X19 − 0.313X20 + 0.726X21 + 0.192X22 + 0.707X23 + 0.34X24 − 0.553X25 + 0.307X26

C3 = 0.629X1 + 0.214X2 + 0.307X3 + 0.052X4 + 0.367X5 + 0.251X6 + 0.007X7 − 0.799X8 + 0.034X9 − 0.455X10 − 0.193X11 + 0.231X12 + 0.322X13 + 0.471X14 − 0.1X15 − 0.893X16 + 0.129X17 + 0.546X18 + 0.723X19 + 0.048X20 + 0.222X21 + 0.076X22 − 0.026X23 − 0.136X24 − 0.757X25 + 0.317X26

C4 = 0.267X1 + 0.319X2 + 0.355X3 − 0.21X4 − 0.425X5 + 0.558X6 + 0.209X7 + 0.17X8 + 0.714X9 − 0.159X10 + 0.009X11 − 0.113X12 + 0.494X13 + 0.097X14 + 0.257X15 + 0.262X16 − 0.718X17 − 0.304X18 + 0.377X19 − 0.594X20 + 0.078X21 − 0.217X22 + 0.486X23 + 0.123X24 + 0.222X25 + 0.046X26

Based on the linear expression formula, the scores of each principal component of the six cultivars of jackfruit were calculated, and then the results of drought tolerance (D) of each variety were calculated by using the affiliation function method and the weighting method of the principal components, as shown in Table 3. The magnitude of the D value indicated the drought tolerance of jackfruit cultivars, and the larger the value, the greater the drought tolerance. The drought tolerance of the six cultivars of jackfruit cultivars was ranked as *A*. ‘Changyou’ > *A*. ‘Haida 4’ > *A*. ‘Haida 2’ > *A*. ‘Haida 3’ > *A*. ‘Haida 1’ > *A*. ‘Siji’. This indicates that *A*. ‘Changyou’ was the most drought-resistant variety and *A*. ‘Siji’ was the least drought-resistant variety according to the available data.

#### 3.4.3. Cluster Analysis of Drought Tolerance

**Figure 9:**
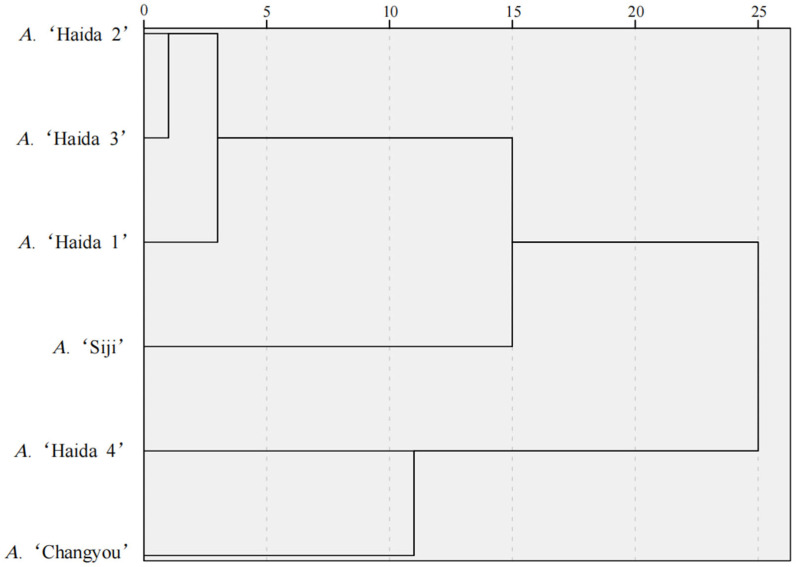
Cluster analysis of drought tolerance in different jackfruit cultivars.

The drought tolerance measures of the six jackfruit resources were standardized by ‘Z-score’ and then clustered (Figure 9). At the genetic distance of 15, the six jackfruit resources could be classified into three major groups. Among them, Class I was the high drought tolerance type, including *A*. ‘Changyou’ and *A*. ‘Haida 4’; Class II was the medium drought tolerance type, including *A*. ‘Haida 2’ and *A*. ‘Haida 3’; and Class III was the low drought tolerance type containing *A*. ‘Haida 1’and *A*. ‘Siji’.

## 4. Discussion

The leaf is the main site of photosynthesis, respiration, and transpiration in plants [22]. Characterization of leaf anatomy is a reliable indicator for studying the drought resistance of plants [23]. Plants with higher drought resistance usually have dry structures such as smaller and thicker leaves, more epidermal hairs, smaller and denser stomata, thicker epidermal cuticles, thicker fenestrated tissues, a higher ratio of fenestrated to spongy parenchyma thickness, and better developed vascular sheaths [24]. The upper and lower leaf epidermis effectively reduced excessive water loss, enhanced the water retention capacity, and reduced damage from exposure to drought stress conditions [25]. A rough leaf epidermis reduces plant transpiration under strong light conditions and helps to reflect light [26]. Plants with small leaf epidermal stomata and high density per unit area are able to respond acutely to environmental changes for rapid stomatal switching [27]. Studies on apricot [7], *Xanthoceras sorbifolium* [28], and *Tectona grandis* [29] found that stomatal density was positively correlated with plant drought tolerance. Fenestrated tissues ensure the transport of water and nutrients as well as the maintenance of photosynthesis [30]. In this study, *A*. ‘Siji’ cultivars with the lowest drought tolerance rating had the thinnest leaves, which were highly significantly lower than the other cultivars. Jackfruit cultivars with high drought tolerance ratings possessed a rougher leaf epidermis and denser stomata, which help in adapting to the drought environment with direct light and regulation of transpiration. The jackfruit cultivar with the highest drought tolerance, *A*. ‘Changyou’, had significantly larger fenestrated parenchyma than the other cultivars and also had the largest ratio of fenestrated to spongy parenchyma thickness. These leaf structural characteristics can be used as an aid to evaluate other jackfruit cultivars for comparative drought resistance.

Drought stress reduces crop chlorophyll concentration, disrupts photosynthesis mechanisms, inhibits growth, and ultimately reduces yield [31,32]. Under drought stress conditions, the membrane system in plant cells, including the membrane structure associated with photosynthesis, is destroyed due to the deficit of moisture, nutrients, and energy, resulting in the disruption of physiological processes [7]. The synthesis of chlorophyll in plants was affected by a variety of factors, including temperature, water, disease, and other stresses [33]. A lack of water in plant leaves affects chlorophyll synthesis, accelerates chlorophyll decomposition, reduces light energy absorption capacity, and inhibits photosynthesis [34,35]. Crop chlorophyll concentration may increase or decrease under drought stress [36,37,38], and the degree of change is closely related to drought tolerance [39,40,41,42]. Drought-tolerant plants usually have higher light saturation points and can adapt to arid environments by regulating photosynthetic structures to improve light energy utilization efficiency [43]. In this study, the chlorophyll concentrations of the leaves of the best drought tolerant cultivars *A*. ‘Changyou’ and *A.* ‘Haida 4’ were lower than those of the other varieties, providing an important guarantee for their good drought tolerance. This may be due to the fact that a lower chlorophyll concentration reduces the rate of photosynthesis in plants, thus helping them to maintain their survival under drought conditions where the energy supply is limited [44]. In the comparison of photosynthetic characteristics, *A*. ‘Siji’, which was the worst evaluated for drought tolerance, was significantly lower than the other cultivars in terms of the net photosynthetic rate, stomatal conductance, and light saturation point. The changes in the chlorophyll concentration and photosynthetic response mechanisms of jackfruit leaves under drought stress need to be demonstrated by more targeted and detailed experiments.

The primary methods for evaluating plant drought tolerance include principal component analysis, membership function analysis, cluster analysis, and gray correlation analysis [45]. These evaluation methods have been widely applied to assess the drought tolerance of various fruit tree species, thereby advancing resistance breeding and the application of fruit tree germplasm resources [10,46]. Jackfruit plants exhibit extremely high heterozygosity and phenotypic diversity, yet systematic studies on the relationship between leaf morphology and photosynthetic characteristics remain limited [47]. Analyzing the association between jackfruit leaf anatomical structure and photosynthetic characteristics can deepen our understanding of tropical fruit trees’ light energy utilization strategies and environmental adaptability, thereby enriching the research content in plant functional ecology. Jackfruit breeding has a relatively long development period. Previous researchers have employed leaf morphological variation and DNA barcoding systems to implement marker-assisted breeding during the nursery stage, thereby enhancing breeding efficiency [48]. By analyzing the differences in photosynthetic characteristics among different cultivars, guidance can be provided for the selection of high-photosynthetic-efficiency cultivars and the development of precise cultivation techniques, thereby improving yield and fruit quality [49]. This study used six jackfruit cultivars bred in China as experimental materials and employed the paraffin parenchyma sectioning method to obtain clear and complete anatomical structure diagrams of the leaves of the six cultivars. Principal component analysis was used to determine the contribution of each indicator to drought tolerance. Based on the weights of each indicator combined with membership functions, drought tolerance metrics were calculated. Finally, cluster analysis was employed for classification, improving the accuracy of the drought tolerance evaluation for jackfruit varieties. However, plant drought tolerance is a trait controlled by multiple genes, influenced not only by its own morphological characteristics and physiological-biochemical properties but also by environmental conditions and cultivation practices. To establish a more comprehensive and reliable evaluation system for jackfruit drought tolerance, it is necessary to expand the sample size and consider multiple factors such as genetic makeup, physiological ecology, and field management practices.

## 5. Conclusions

In this study, we compared the leaf structural characteristics, chlorophyll concentration, and photosynthetic parameters of six jackfruit cultivars and conducted a comprehensive evaluation of their drought tolerance. From 26 leaf indicators, the following were selected as the primary evaluation criteria for jackfruit drought tolerance: leaf width, lower epidermal cell thickness, transpiration rate, chlorophyll concentration, leaf parenchyma structural density, net photosynthetic rate, stomatal conductance, spongy parenchyma thickness, leaf parenchyma structural porosity, stomatal density, instantaneous water use efficiency, thick-walled parenchyma thickness, and stomatal width. Comprehensive analysis revealed that the jackfruit cultivar with the highest drought tolerance score was *A*. ‘Changyou’, while the cultivar with the lowest drought tolerance score was *A*. ‘Siji’. In this study, jackfruit cultivars with high drought resistance had significantly thicker palisade parenchyma than other cultivars, a rougher leaf epidermis, and more densely spaced stomata, with the chlorophyll concentration being significantly lower than that of the other cultivars. Jackfruit cultivars with the lowest drought resistance had a significantly lower net photosynthetic rate, transpiration rate, stomatal conductance, and light saturation point than other cultivars. Future studies could evaluate more jackfruit germplasm resources to screen for additional varieties with superior drought resistance and establish a more systematic evaluation system for jackfruit drought resistance. Additionally, further exploration of the intrinsic relationships between drought resistance and leaf structural characteristics, chlorophyll concentration, and photosynthetic parameters could reveal the physiological mechanisms underlying drought resistance, providing technical support for drought-resistant jackfruit cultivation. Nevertheless, the results of this study provide clear microscopic structural images of leaves from different jackfruit cultivars, offering key indicators for evaluating the drought tolerance of jackfruit germplasm resources. This study contributes to the evaluation of drought tolerance in jackfruit germplasm resources and provides data support for screening and breeding high-quality jackfruit cultivars with superior drought tolerance.

## Figures and Tables

**Table 1:** Experimental jackfruit variety information [17].

Number	Variety	Country of Origin	Variety Approval Number
1	*A*. ‘Haida 1’	Donghai Island, Zhanjiang	Guangdong 2013004
2	*A*. ‘Haida 2’	Changshan Town, Lianjiang, Zhanjiang	Guangdong 2014009
3	*A*. ‘Haida 3’	Nanshan Town, Xuwen, Zhanjiang	Guangdong 2014003
4	*A*. ‘Haida 4’	Nanshan Town, Xuwen, Zhanjiang	Guangdong 2023006
5	*A*. ‘Siji’	Dongan Town, Gaozhou, Maoming	Guangdong 2009019
6	*A*. ‘Changyou’	Fruit Tree Research Institute, Maoming	Guangdong 2008001

**Table 2:** Matrix of component score coefficients.

Indexes	Principal Component Coefficient			
	C1	C2	C3	C4
X1	0.309	−0.037	0.629	0.267
X2	0.809	0.432	0.214	0.319
X3	0.725	0.407	0.307	0.355
X4	−0.743	−0.582	0.052	−0.21
X5	0.462	0.677	0.367	−0.425
X6	−0.748	−0.258	0.251	0.558
X7	0.774	−0.586	0.007	0.209
X8	0.373	0.375	−0.799	0.17
X9	0.23	−0.652	0.034	0.714
X10	0.625	0.341	−0.455	−0.159
X11	0.488	0.607	−0.193	0.009
X12	−0.846	0.3	0.231	−0.113
X13	0.735	−0.327	0.322	0.494
X14	0.489	−0.688	0.471	0.097
X15	0.608	−0.739	−0.1	0.257
X16	0.228	0.19	−0.893	0.262
X17	0.394	−0.478	0.129	−0.718
X18	0.757	−0.19	0.546	−0.304
X19	−0.452	0.357	0.723	0.377
X20	0.74	−0.313	0.048	−0.594
X21	0.636	0.726	0.222	0.078
X22	0.916	0.192	0.076	−0.217
X23	0.5	0.707	−0.026	0.486
X24	−0.902	0.34	−0.136	0.123
X25	0.115	−0.553	−0.757	0.222
X26	−0.896	0.307	0.317	0.046
Characteristic root	10.539	5.934	4.393	3.302
Contribution rate%	40.533	22.824	16.898	12.7
Cumulative contribution%	40.533	63.357	80.255	92.955

**Table 3:** Evaluation of drought tolerance in different jackfruit cultivars.

Cultivars	Principal Component Score				Value of the Affiliation Function				Consolidated Assessed Value	Rankings
	C1	C2	C3	C4	C1	C2	C3	C4	Comprehensive Evaluation Value (D)	
*A*. ‘Haida 1’	−12.62	4.55	−0.16	−2.87	0.00	1.00	0.57	0.00	0.32	5
*A*. ‘Haida 2’	−3.46	3.10	0.59	6.41	0.35	0.90	0.63	1.00	0.58	3
*A*. ‘Haida 3’	−2.38	2.36	4.42	−1.98	0.39	0.86	0.96	0.10	0.53	4
*A*. ‘Haida 4’	11.66	4.02	−6.66	−1.03	0.92	0.96	0.00	0.20	0.62	2
*A*. ‘Siji’	−7.07	−10.58	−3.01	−0.03	0.21	0.00	0.32	0.31	0.18	6
*A*. ‘Changyou’	13.87	−3.45	4.82	−0.50	1.00	0.47	1.00	0.26	0.71	1

## Data Availability

The raw data supporting the conclusions of this article will be made available by the authors on request.

## Supplementary Materials

The following supporting information can be downloaded at https://www.mdpi.com/article/10.3390/life15091346/s1. Figure S1: Daily variation in net photosynthetic rate of different jackfruit cultivars. A: *A*. ‘Haida 1’; B: *A*. ‘Haida 2’; C: *A*. ‘Haida 3’; D: *A*. ‘Haida 4’; E: *A*. ‘Siji’; F: *A*. ‘Changyou’. Different lowercase letters indicate significant differences (*p* < 0.05). Figure S2: Daily variation in transpiration rates of different jackfruit cultivars. A: *A*. ‘Haida 1’; B: *A*. ‘Haida 2’; C: *A*. ‘Haida 3’; D: *A*. ‘Haida 4’; E: *A*. ‘Siji’; F: *A*. ‘Changyou’. Different lowercase letters indicate significant differences (*p* < 0.05). Figure S3: Daily changes in stomatal conductance of different jackfruit cultivars. A: *A*. ‘Haida 1’; B: *A*. ‘Haida 2’; C: *A*. ‘Haida 3’; D: *A*. ‘Haida 4’; E: *A*. ‘Siji’; F: *A*. ‘Changyou’. Different lowercase letters indicate significant differences (*p* < 0.05). Figure S4: Intercellular CO2 daily changes in different jackfruit cultivars. A: *A*. ‘Haida 1’; B: *A*. ‘Haida 2’; C: *A*. ‘Haida 3’; D: *A*. ‘Haida 4’; E: *A*. ‘Siji’; F: *A*. ‘Changyou’. Different lowercase letters indicate significant differences (*p* < 0.05). Table S1: Comparison of anatomical structures of jackfruit leaves from different cultivars.
