# Supplementary material for: Comprehensive Evaluation of Leaf Structure, Photosynthetic Characteristics, and Drought Resistance in Six Jackfruit (Artocarpus heterophyllus) Cultivars
Source: Life (Basel). 2025 Aug 26;15(9):1346. doi: 10.3390/life15091346 (PMC12470841; doi:10.3390/life15091346)
Supplement: Supplementary file 1 [file life-15-01346-s001.zip › life-3770907-supplementary.pdf]

Supplementary pictures:

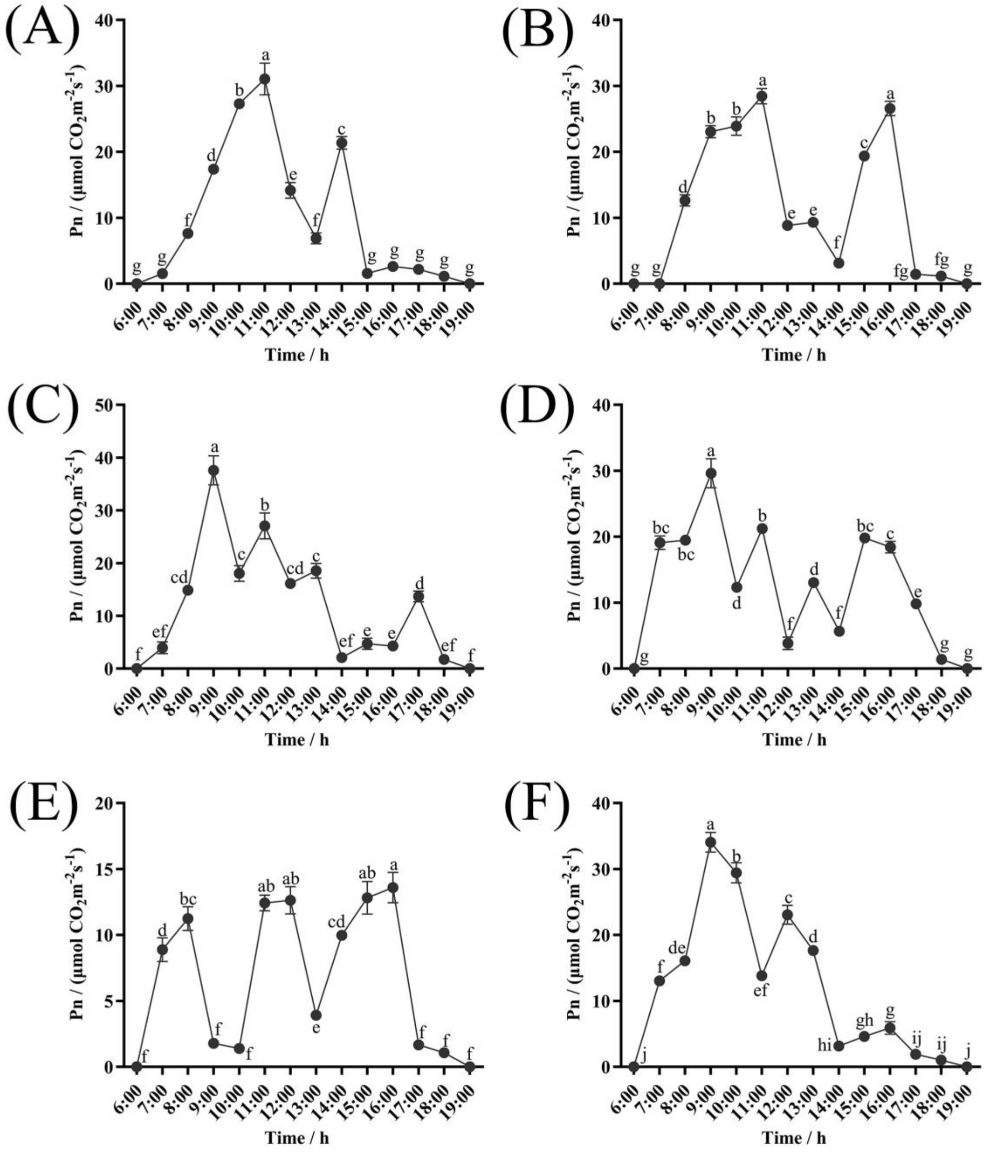

Supplementary Figure 1. Daily variation in net photosynthetic rate of different jackfruit cultivars. A：*A.*'Haida 1'; B：*A.*'Haida 2'; C: *A.*'Haida 3'; D: *A.*'Haida 4'; E:*A.*'Siji'; F: *A.* 'Changyou'; Different lowercase letters indicate significant differences ($p < 0.05$).

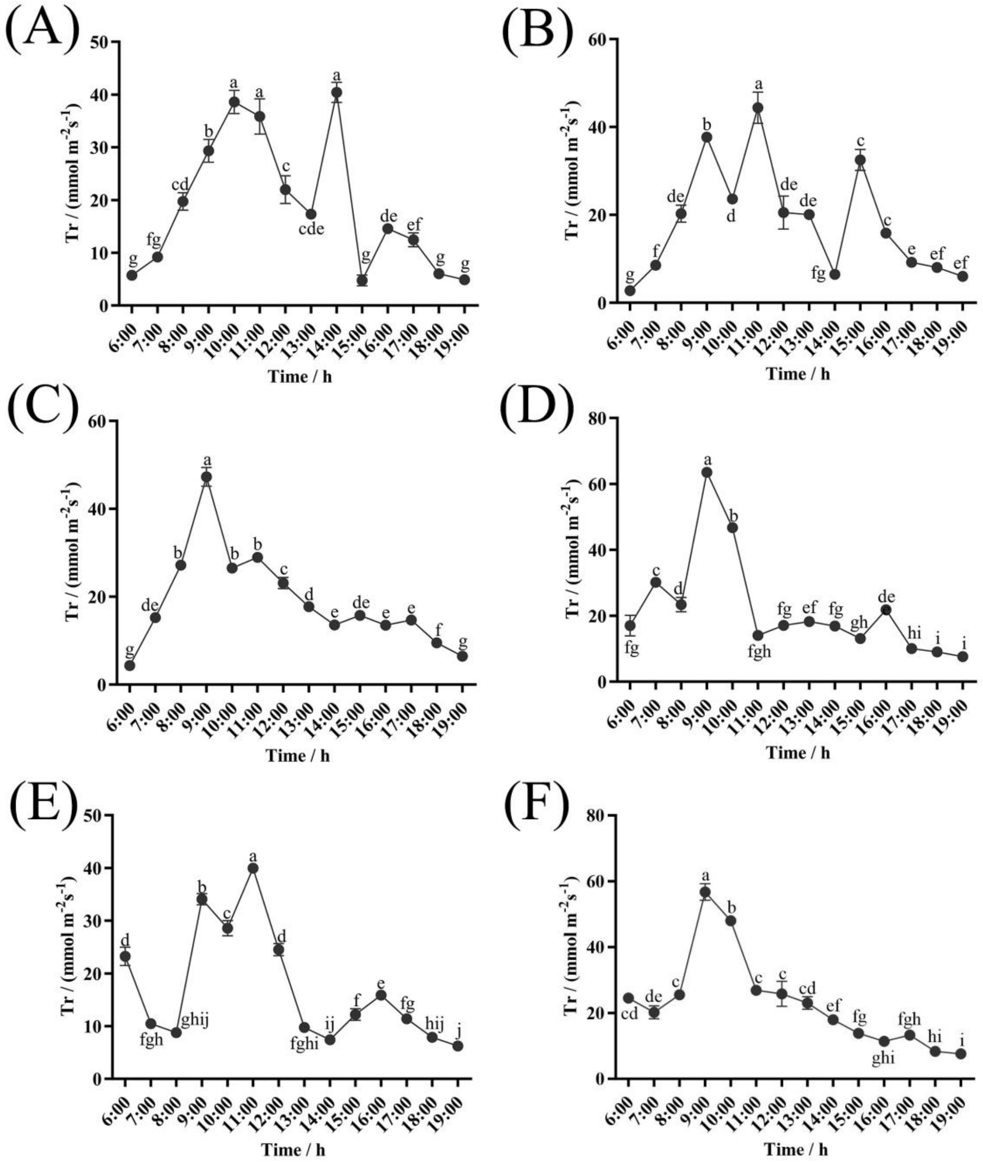

Figure 2. Supplementary Figure 2. Daily variation in transpiration rates of different jackfruit cultivars. A：*A.*‘Haida 1’; B：*A.*‘Haida 2’; C: *A.*‘Haida 3’; D: *A.*‘Haida 4’; E:*A.*‘Siji’; F: *A.* ‘Changyou’; Different lowercase letters indicate significant differences ($p < 0.05$).

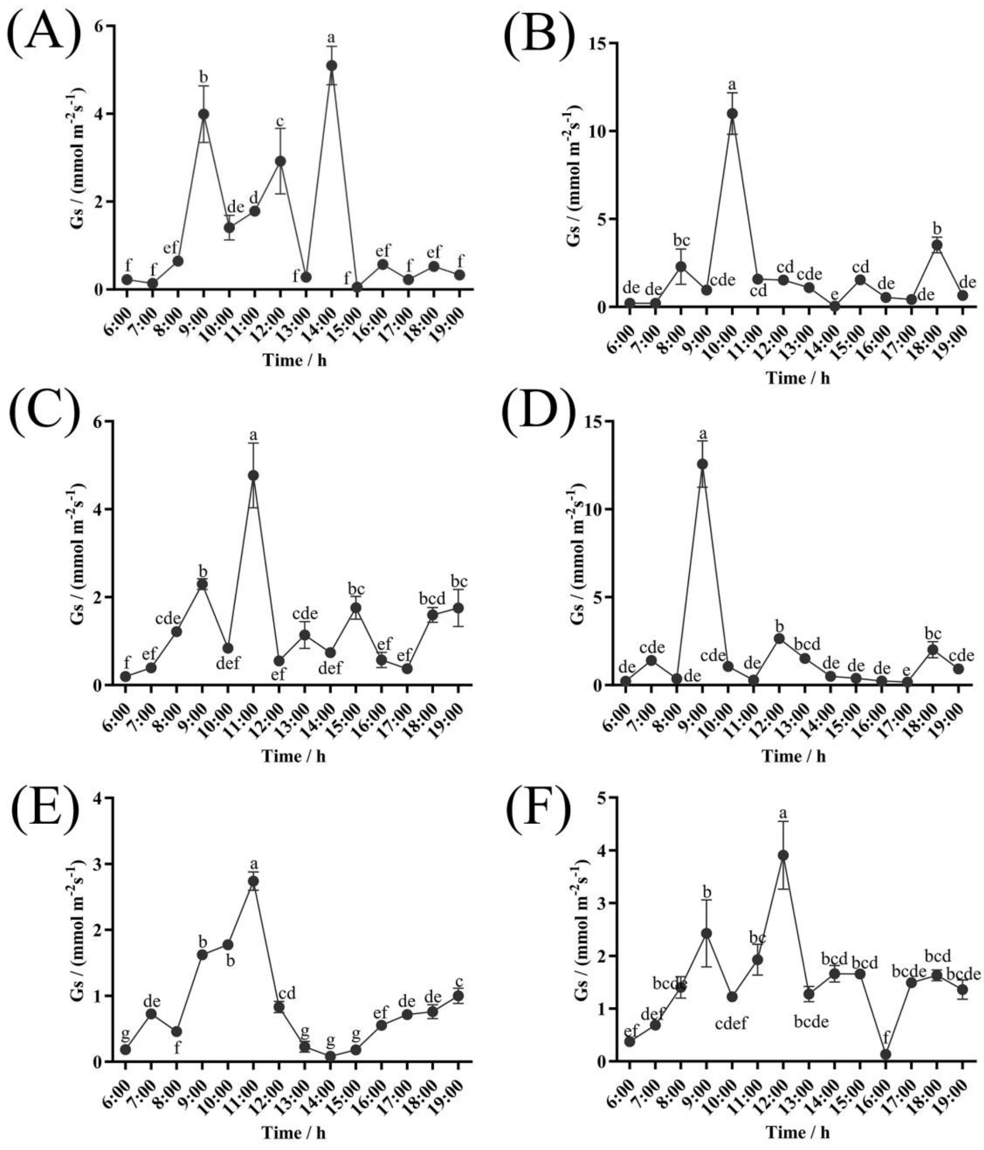

Supplementary Figure 3. Daily changes in stomatal conductance of different jackfruit cultivars. A ：*A*.'Haida 1'; B：*A*.'Haida 2'; C: *A*.'Haida 3'; D: *A*.'Haida 4'; E:*A*.'Siji'; F: *A*. 'Changyou'; Different lowercase letters indicate significant differences ($p < 0.05$).

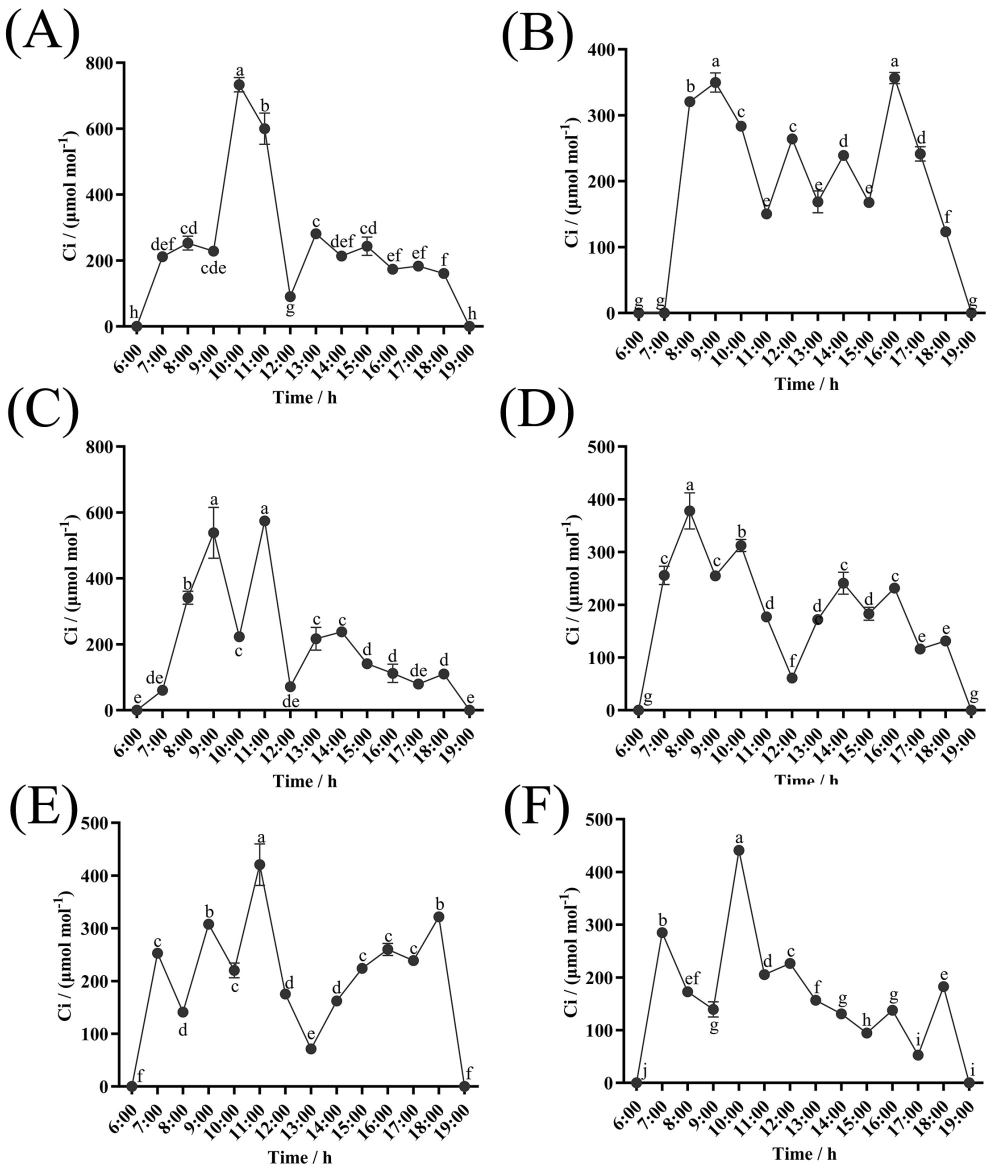

Supplementary Figure 4. Intercellular CO_2 daily changes in different jackfruit cultivars. A：*A.*'Haida 1'; B：*A.*'Haida 2'; C: *A.*'Haida 3'; D: *A.*'Haida 4'; E:*A.*'Siji'; F: *A.* 'Changyou'; Different lowercase letters indicate significant differences ($p < 0.05$).

Supplementary Table 1. Comparison of anatomical structures of jackfruit leaves from different cultivars.

Species	Leaf thickness (LT)/mm	Vessel diameter (VD)/μm	Palisade parenchyma thickness (TP)/μm	Sponge parenchyma thickness (TS)/μm	Thick wall thickness (TW)/μm	Thickness of upper epidermal cells (TU)/μm	Thickness of the upper epidermal thick-walled parenchyma (UC)/μm	Thickness of lower epidermal cells (TL)/μm	Lower epidermal thick-walled parenchyma thickness (LC)/μm	parenchyma Ratio of Palisade to Spongy（TRPS）	Cell Tightness Ratio（CTR）/%	Spongy Mesophyll Ratio（SR)/%
A.'Haida 1'	0.41±0.01 abA	27.28±1.35 abABC	32.04±0.81 eE	179.69±4.76 cdCD	31.44±1.72 cC	5.71±0.25 bB	3.78±0.13 abA	5.59±0.19 aAB	2.81±0.09 dE	0.18±0.01 dE	8.03±0.26 dD	44.8±1.24 cC

A.'Haida 2'	0.39±0.01 bA	30.48±1.54 aA	51.28±1.37 cC	218.47±9.31 bB	53.48±2.04 aA	6.03±0.27 bB	3.62±0.08 bA	5.04±0.25 bCD	3.85±0.16 bB	0.25±0.01 cC	13.09±0.38 bB	56.03±2.65 bB
A.'Haida 3'	0.43±0.01 aA	26.7±1.24 abABC	42.8±1.22 dD	183.58±7.92 cdCD	40.11±2.57 bB	6.58±0.27 bB	3.14±0.1 cB	5.81±0.25 aD	3.37±0.1 cC	0.24±0.01 cCD	10.23±0.39 cC	43.56±2.01 cdC
A.'Haida 4'	0.42±0.01 abA	21.89±1.23 dC	58.77±2.14 bB	293.04±3.69 aA	41.09±1.79 bB	7.43±0.45 aA	3.97±0.1 aA	4.1±0.15 cE	3.52±0.12 cBC	0.2±0.01 dD	14.29±0.61 bB	71.09±1.52 aA
A.'Siji'	0.36±0 cB	29.13±1.58 aAB	60.9±2.28 bB	190.66±4.87 cC	53.82±2.55 aA	5.93±0.2 bB	2.98±0.09 cB	4.83±0.13 bA	3.48±0.09 cBC	0.33±0.02 bB	17.14±0.74 aA	53.4±1.46 bB
A.'Changyou'	0.42±0.01 aA	24.67±1.6 cdBC	75.23±1.46 aA	166.02±2.63 dD	49.47±2.35 aA	6.09±0.19 bB	3.8±0.14 abA	3.77±0.09 cE	4.3±0.13 aA	0.46±0.01 aA	17.97±0.5 aA	39.6±0.91 dC
